# Telomerase as a Possible Candidate Targeting Therapy in Different Breast Cancer Cell Lines

**DOI:** 10.31557/APJCP.2020.21.8.2243

**Published:** 2020-08

**Authors:** Salma Aboelela, Abeer Ashmawy, Sabry Shaarawy, Mohammed EL- Hefny, Amina Medhat

**Affiliations:** 1 *Electron Microscopy Unit, Cancer Biology Department, National Cancer Institute, Cairo University, Cairo Egypt. *; 2 *Biochemistry unit, Cancer Biology Department, National Cancer Institute, Cairo University, Cairo, Egypt. *; 3 *Biochemistry unit, Cancer Biology Department, National Cancer Institute, Cairo University, Cairo Egypt. Department of Medical Genetics, Umm Al-Qura University, Alqunfudah, KSA. *; 4 *Biochemistry Department, Faculty of Science, Ain-Shams University, Cairo Egypt. *

**Keywords:** Telomerase, siRNA, Doxorubicin, breast cancer

## Abstract

**Background:**

Telomerase activity is up regulated in most breast cancer subtypes but not in the adjacent normal tissues. Thus, it is a promising target for anticancer therapy. The present work investigated the effects of telomerase inhibition by siRNA on breast cancer cell lines and studied the feasibility of whether the combined effect of doxorubicin with siRNA treatment on breast cancer cells potentiates a rapid cellular response to the cytotoxic effect of chemotherapy.

**Methods:**

This study was performed on Luminal A (MCF-7), triple negative (MDA-MB-468), and HER-2/neu (SKBR-3) human breast cancer cell lines, wherein telomerase activity inhibition by hTERT siRNA and doxorubicin was detected by measuring telomerase activity using Telomeric Repeat Amplification Protocol (TRAP assay), assessing cell viability through MTT assay, and evaluating apoptosis through scanning electron microscopy (SEM) and through estimating caspase-3 and -8 activities using enzyme-linked immunosorbent assay (ELISA).

**Results:**

In the present study, hTERT siRNA effectively reduced telomerase activity and cell viability to more than 90% and 60%, respectively, in most breast cancer cell lines within 72 hours after transfection. The combination of hTERT siRNA and doxorubicin showed a cumulative effect compared with either treatment alone (P < 0.05). Meanwhile, SEM demonstrated apoptotic morphologic cell changes.

**Conclusion:**

Telomerase inhibition is a promising strategy for the effective treatment of breast cancer. When used in combination with doxorubicin, it could potentiate the cytotoxic effect of the drug on breast cancer cells.

## Introduction

Telomeres are repetitive, non-coding DNA sequences located at the ends of each chromosome (Louzon et al., 2019). Telomerase is a ribonucleoprotein enzyme, active in 80% - 90% of malignancies, and its function is to maintain chromosomal telomeres (Arndt and MacKenzie, 2016). The telomerase complex consists of two components, namely, the catalytic sub-unit human telomerase reverse transcriptase (hTERT) and telomeric RNA component (hTERC) (Peska and Garcia, 2020). Telomerase is inactive in most normal human cells due to the strict hTERT transcriptional repression; however its activation is considered a prior step for malignant transformation of human cell (Both et al., 2017). Moreover, hTERT has other evident biological activities beside its function in telomere lengthening. For instance, it protects cancer cells from apoptosis induced by chemotherapeutic drugs. Additionally, high telomerase expression makes cancer cells resistant to chemotherapy and radiotherapy (Saretzki, 2015). RNA interference (RNAi) is a post-transcriptional gene-silencing mechanism. Furthermore, small interference RNA (siRNA) are small double-stranded RNAs of approximately 22 nucleotides that participate in RNAi. An advantage of siRNA is that they are not integrated into DNA, thus, they do not permanently modify the genome (Huynh et al., 2018). Breast cancer (BC) is the most commonly diagnosed cancer and the leading cause of cancer deaths among women in over 100 countries (Bray et al., 2018). It is a heterogeneous group of pathologic entities, and three subtypes of breast tumors that differ in biologic behavior were identified through immunohistochemistry techniques: hormone receptor-positive, human epidermal receptor (HER2/neu) positive, and triple negative breast cancers (TNBCs), and each of these requires a specific management approach (Bianchini et al., 2016). In BC patients, a significant association was observed between telomerase-positive invading breast carcinomas and lymphovascular invasion, which is a crucial step in breast tumor metastasis and a predictor of survival, which makes telomerase a helpful prognostic marker. Accordingly, the link between genetic changes associated with BC and telomerase activity remains an imperative research area nowadays (Jitariu et al., 2017). Doxorubicin, an anthracycline, is a powerful first-line chemotherapeutic drug with cytotoxic activity used in BC treatment. Doxorubicin utility is constrained by its toxicity due to cumulative doses, and its toxicity can be minimized, if it is used in combination with a more tumor-specific treatment that aids in dose reduction (Wang et al., 2019). In this study, we hypothesized the synergism between doxorubicin and restrained telomerase activity by siRNA, causing enhanced cancer cells sensitivity to treatment in several BC cell lines.

## Materials and Methods

*Materials*

*Cell Culture*

The study was performed on three human BC cell lines: MCF-7 (Luminal A), MDA-MB-468 (triple-negative breast cancer), and SKBR-3 (HER2 positive). All cell lines were obtained from the American Type Culture Collection (ATCC, Washington, DC, USA) and maintained by serial sub-culturing at University Medical Center, Hamburg-Eppindorf, Germany, then sub-cultured and passaged at the National Cancer Institute, Cairo University, Egypt. The characterization method used by ATCC is the short tandem repeat (STR) DNA profile. Cells were grown in Dulbecco’s Modified Eagle’s Medium (DMEM) supplemented with 10% fetal bovine serum (FBS) and 1% antibiotic-antimycotic drug, then maintained in monolayer culture at 37°C and 5% CO_2_ atmosphere. All chemicals were purchased from (Biowest, France).

*Methods*

*Determination of IC*_50_

Cancer cells cytotoxicity for doxorubicin (Doxorubicin HCL, 10 mg, 1 vial, 5ml) (Adricin) was measured using the sulforhodamine B (SRB) assay using a previously described method (Skehan et al., 1990). After 48 hours of incubation with different concentrations of doxorubicin (0, 5, 12.5, 25 and 50 µg/ml), the cells were fixed with 10% (wt/vol) trichloroacetic acid and stained for 30 min. The excess dye was removed by washing repeatedly with 1% (vol/vol) acetic acid. The number of viable cells was directly proportional to the protein-bound dye formation that was then solubilized with tris base solution (10 mM), pH 10.5, and measured through a fluorometric assay ELISA at 570 nm (Bio-Rad, Hercules, CA, USA; Microplate Reader). Two separate experiments were performed in triplicates, and IC50 was assessed from the survival curves. 

*Transfection*

Each cell line was presented in four groups: Group 1) cancer cells without any treatment serving as control group, Group 2) cancer cells transfected with siRNA, Group 3) cancer cells treated with IC_50_ dose of doxorubicin, and Group 4) combined treatment of cancer cells transfected with siRNA and the IC_50_ dose of doxorubicin. The N-TER nanoparticle siRNA transfection system (Sigma - Aldrich, Germany) was used for siRNA delivery. According to the manufacturer’s protocol, the cells were plated in 6-well plates at a seeding density of 2.4 × 10^5^ cells/well the day before transfection, and simultaneously transfected alone or in combination with siRNA 50 nmol (Eurofins Genomics, Germany). The cells were cultured in serum and antibiotic-antimycotic-free DMEM and incubated for 4 hours with siRNA. Then, 2 × serum-containing medium with 20% FBS was added to all wells and left overnight. The following day, the transfection medium was changed with fresh medium without antibiotic-antimycotic and the selected groups were treated with the estimated IC_50 _dose of doxorubicin, and the plates were incubated for 48 hours. The siRNA duplex targeting mRNA of hTERT termed siRNA#1 was selected based on our previous study (Ashmawy et al., 2011), synthesized, and purified (Eurofins Genomics, Germany), with the length of 19 nucleotides as follows: Sense sequence: 5'GUGUCUGUGCCCGGGAGAAdTdT3' Anti-sense sequence: 5'UUCUCCCGGGCACAGACACdTdT3' The selected sequences were submitted to BLAST (http://www.ncbi.nlm.nih.gov/blast/) to ensure that the selected genes were specifically targeted. 

*Telomerase activity assay*

Telomerase activity was determined through TRAP assay using TRAPeze Kit RT (Millipore, USA), according to the manufacturer’s protocol. Protein concentration was measured through NanoDrop One 10 - 750 ng/µL (Thermo Fisher Scientific). For the TRAP reaction, 2 µL of cell extract was added to a final volume of 25 µL of the reaction mixture. PCR was then performed as follows: Telomerase activity (30 min, 30°C), Taq activation (2 min, 95°C), and product amplification for 45 cycles (94 °C for 15 s, 59°C for 60 s, and 45°C for 10 s) using a Real-Time thermal cycler (Applied Biosystem Viia-7, USA). Three independent experiments were performed.

*MTT Assay for measuring cell viability*

The cells were plated in 96-well plates (5× 10^3^ cells/well) with 100 µL of DMEM in each well. After 24 hours of transfection, the appropriate wells were treated with the specific IC_50_ dose of doxorubicin specified for each cell line, incubated for another 48 hours, then the cell viability was detected using MTT Cell Proliferation Assay kit (Berridge et al., 2005). Absorbance of each sample was measured at 570 nm using a microplate reader. Cell viability was expressed as a percentage, assigning the viability of control cells as 100%. Three independent experiments were performed.

*Caspase-3 and -8 activity assays*

Quantitative detection of human Caspase-3 and -8 was performed according to manufacturer’s protocol (Affymetrix eBioscience, Austria and Biospes, China), respectively, through ELISA. The absorbance of each well was measured at 450 nm using a microplate reader in three independent experiments.

*Scanning Electron Microscopy (SEM)*

The cells were collected, fixed with glutaraldehyde 2.5% for 3 hours then washed with PBS (0.1 M) for 10 minutes and incubated with PBS overnight. The cells were then incubated for 1.5 hours with osmium tetroxide (OsO4) followed by dehydration with 50% and 70% alcohol, respectively (Hayat, 1981). Morphologic changes were examined through SEM (Inspect S; Holland) at the electron microscopy unit of the Theodor Bilharz Research Institute. 

*Statistical analysis*

Statistical analysis of the difference between means was carried out using one-way analysis of variance (ANOVA). In case of a significant F-ratio, post hoc least significant difference (LSD) test for multiple comparisons was used to evaluate the statistical significance between treated groups at P < 0.05. Data are presented as mean ± SD of three independent experiments. All statistical analyses were done using the Statistical Package for Social Science (SPSS) software version 22.0 (SPSS Inc., Chicago, IL, USA). Statistical analysis of doxorubicin IC50 results was performed using one-way ANOVA followed by Tukey’s multiple comparison test at significance P < 0.05 using the GraphPad Prism5.0 software (GraphPad Software, Inc., San Diego, California, USA). The results were presented as mean ± SD of two separate experiments performed in triplicates. 

## Results

*Detection of Inhibitory Concentration 50 (IC*_50_ *) values through SRB Assay*

**Figure 1:**
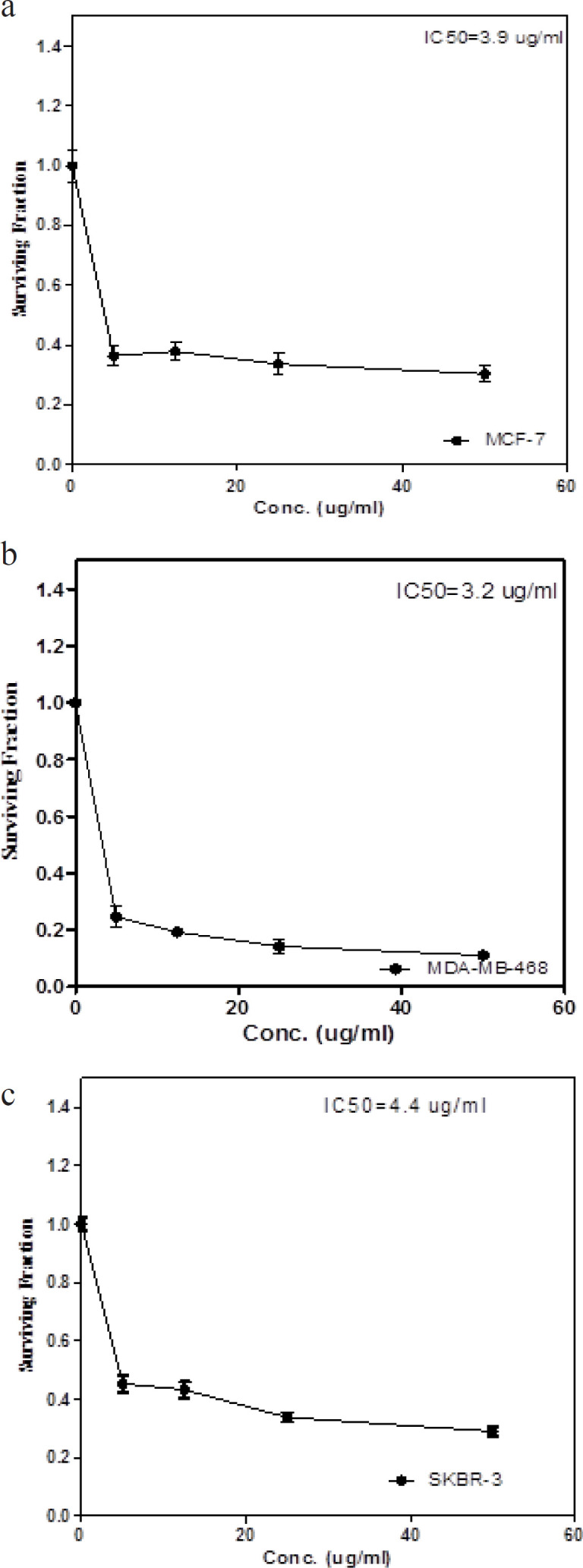
Surviving Fraction Curves for IC_50_ of Doxorubicin for the Three Subtypes of Breast Cancer Cell Lines; a) MCF-7 cell line (Luminal A), b) MDA-MB-468 cell line (Triple negative) and c) SKBR-3 cell line (HER-2/neu). The results were presented as the mean ± SD of 2 separate experiments. The statistical significance of the results was analyzed using one way ANOVA followed by Tukey’s multiple comparison test, data are significant at P < 0.05

Our data indicated the surviving fraction of MCF-7, MDA-MB-468, and SKBR-3 cells treated with 0, 5, 12.5, 25, and 50 µg/ml doxorubicin for 48 hours. The concentrations at which cell growth was inhibited by IC50 after a 48 hour treatment period with doxorubicin were 3.9 ± 0.141, 3.2 ± 0.14, and 4.4 ± 0.142 µg/ml, for MCF-7, MDA-MB-468, and SKBR-3, respectively (P < 0.01), compared with the control group, as shown in Figure 1 (a, b, and c).

*Effects of different treatments on telomerase activity*

**Figure 2:**
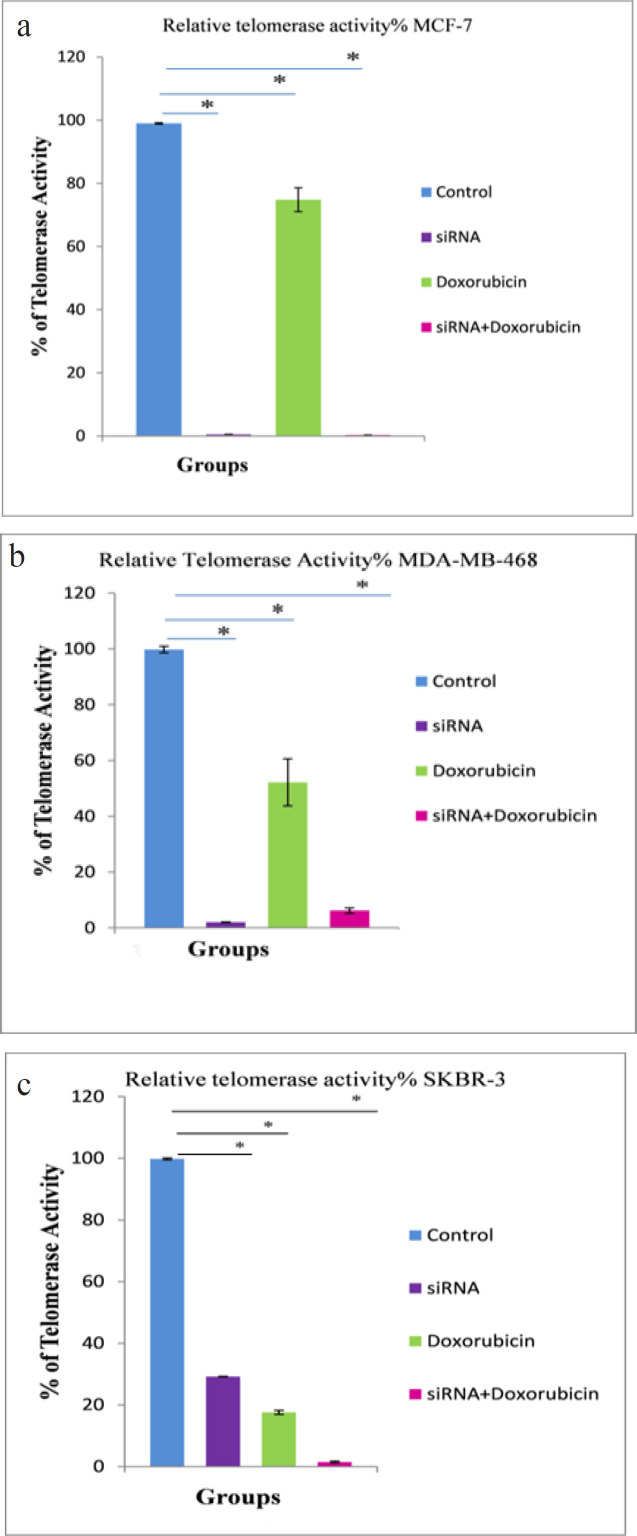
Relative Telomerase Activity Percentage Curves for a) Luminal A (MCF-7), b) Triple negative (MDA-MB-468) and c) HER-2/neu (SKBR-3) cell lines. Telomerase activity was detected by TRAP assay via real-time PCR in the three cell lines 72 hours after exposure to hTERT siRNA and doxorubicin. The difference between means was carried out using one-way analysis of variance (ANOVA). Data are shown as mean ± SD (error bar) of 3 experiments, * data are significant at P < 0.05

In Figure 2, the MCF-7 BC cell line (Figure 2a) showed a significant inhibition of telomerase activity (P < 0.001) in the combined treated group (siRNA + Dox.), and the siRNA-transfected group showed a significant reduction in telomerase activity (P < 0.001), in which 0.53% ± 0% of telomerase activity was only detected, however, the doxorubicin-treated group revealed a significant reduction in telomerase activity to 74.83% ± 3.8% (P < 0.001), compared with the control group. In the MDA-MB-468 BC cell line (Figure 2b), telomerase activity was significantly reduced to 2.0% ± 0.10% and 6.20% ± 0.96% (P < 0.001) in the siRNA-transfected and combined treated groups, respectively, and it was also significantly reduced to only 52.13% ± 8.46% (P < 0.001) in the doxorubicin-treated group, compared with the control group. In the SKBR-3 BC cell line (Figure 2c), telomerase activity was significantly reduced to 1.50% ± 0.26 % (P < 0.001) in the combined treated group, compared with the control group. 

*Effects of different treatments on cell viability*

**Figure 3:**
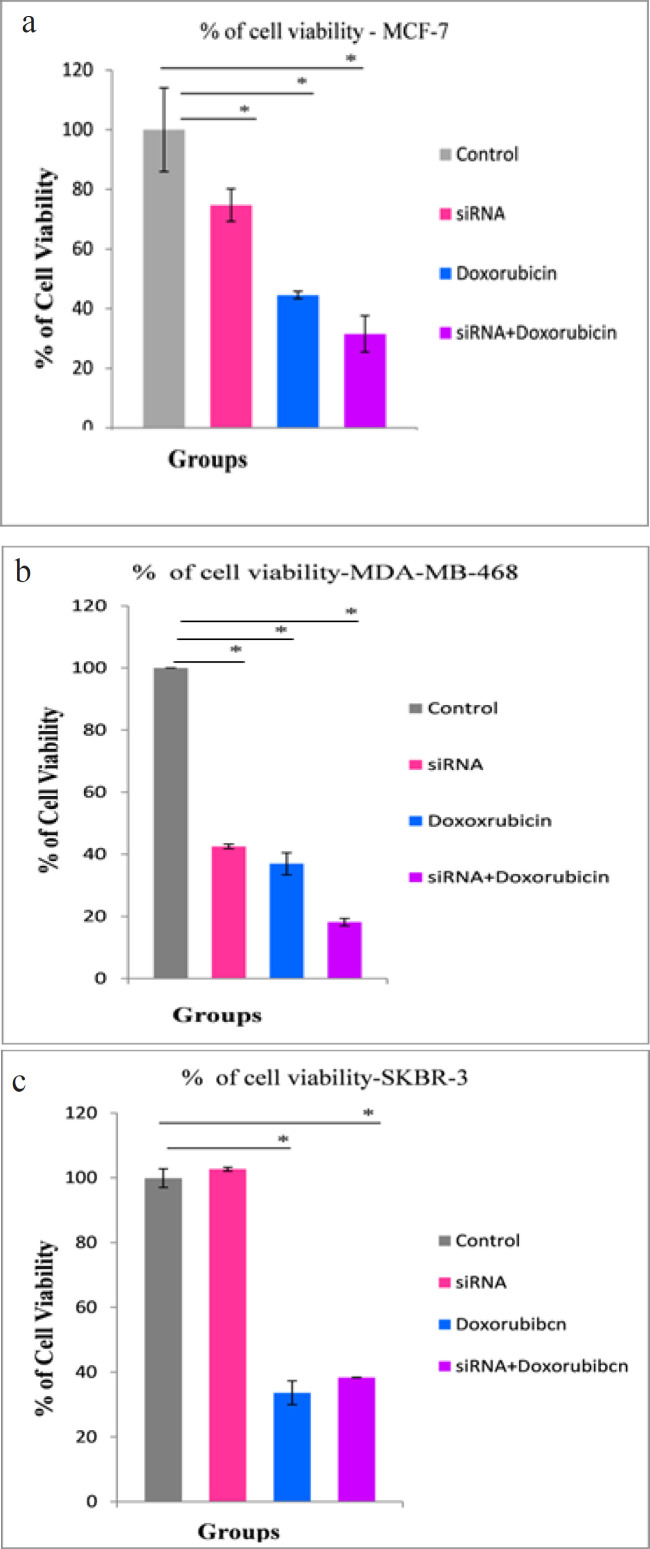
Percentage of Cell Viability in Breast Cancer Cell Lines Measured by MTT Assay in a) MCF-7 b) MDA-MB-468 and c) SKBR-3 cell lines 72 hours after exposure to hTERT siRNA and Doxorubicin. The difference between means was carried out using one-way analysis of variance (ANOVA). Data are shown as mean ± SD (error bar) of 3 experiments, * data are significant at P < 0.05

In Figure 3, the cell viability of MCF-7 cell line, was significantly reduced to 31.50% ± 6.08% in the combined treated group (P < 0.001), however, there was a significant reduction in the viability of the siRNA-transfected group to only 74.73% ± 5.46% (P < 0.04), compared with the control group (Figure 3a). In the MDA-MB-468 cell line, there was a significant reduction in the cell viability of the combined treated group to 18.13% ± 1.19% (P < 0.001), and the doxorubicin-treated and hTERT siRNA-transfected groups, showed a significant reduction in cell viability to 36.96% ± 3.55% and 42.50% ± 0.75% (P < 0.001), respectively, compared with the control group (Fig.3b). In the SKBR-3 cell line, there was an insignificant change in the viability of the siRNA-transfected group, compared with the control group, wherein cell viability increased to 102.66% ± 0.57% in the siRNA-transfected group. The doxorubicin-treated and the combined treated groups showed a significant reduction in cell viability to 33.63% ± 3.66% and 38.33% ± 0.057% (P < 0.001), respectively, compared with the control group (Figure 3c). 

*Effects of different treatments on Caspase-8 activity*

**Figure 4:**
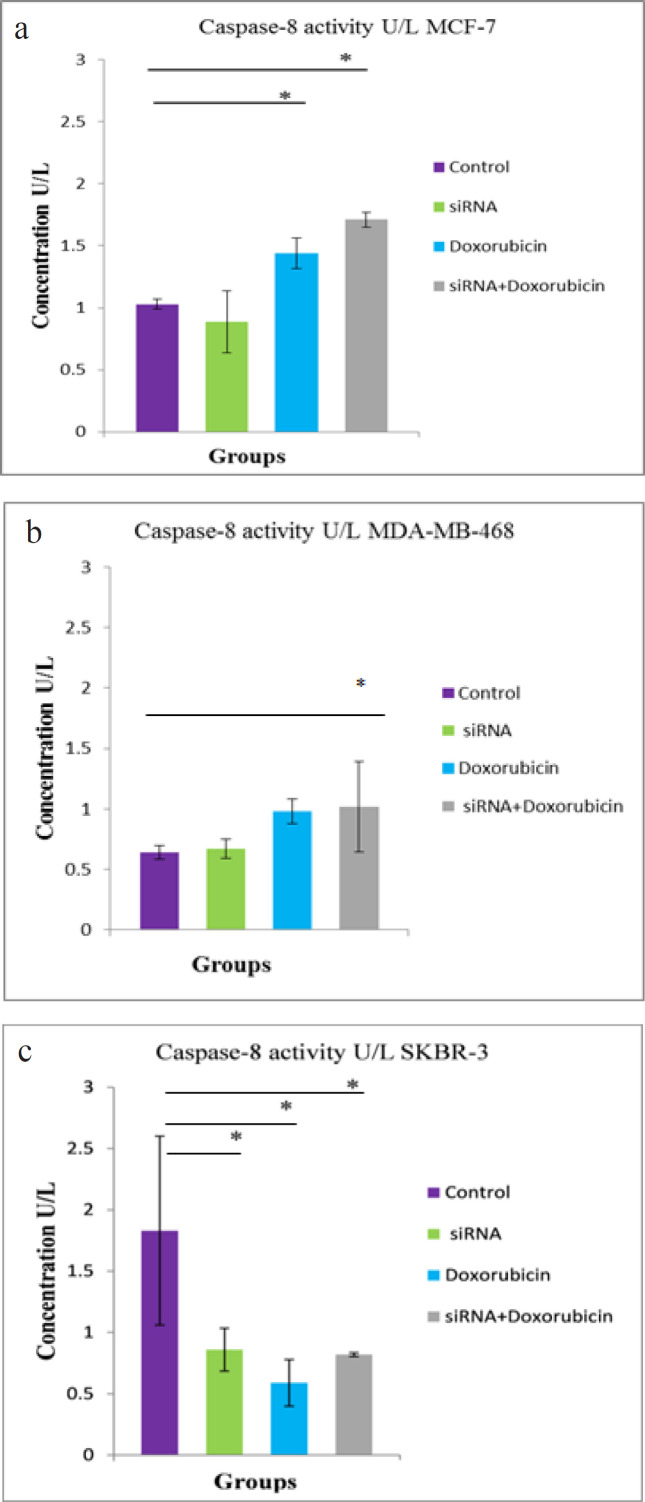
Caspase-8 Activity in a) MCF-7 b) MDA-MB-468 and c) SKBR-3 cell lines 72 hours after exposure to hTERT siRNA and doxorubicin. The difference between means was carried out using one-way analysis of variance (ANOVA). Data are shown as mean ± SD (error bar) of 3 experiments, * data are significant at P < 0.05

In Figure 4, the combined treatment and doxorubicin-treated groups showed a significant increase in caspase-8 activity (P < 0.001 and P < 0.008, respectively) in the MCF-7 cell line, compared with the control group (Figure 4a). In the MDA-MB-468 cell line, a significant increase (P < 0.04) in caspase-8 activity was observed only in the combined treated group, compared with the control group (Figure 4b). In the SKBR-3 cell line, a significant reduction was noticed in caspase-8 activity in the hTERT siRNA-transfected, doxorubicin-treated, and combined treated groups (P < 0.019, P < 0.006, and P < 0.016, respectively), compared with the control group (Figure 4c).

*Effects of different treatments on Caspase-3 activity*

**Figure 5:**
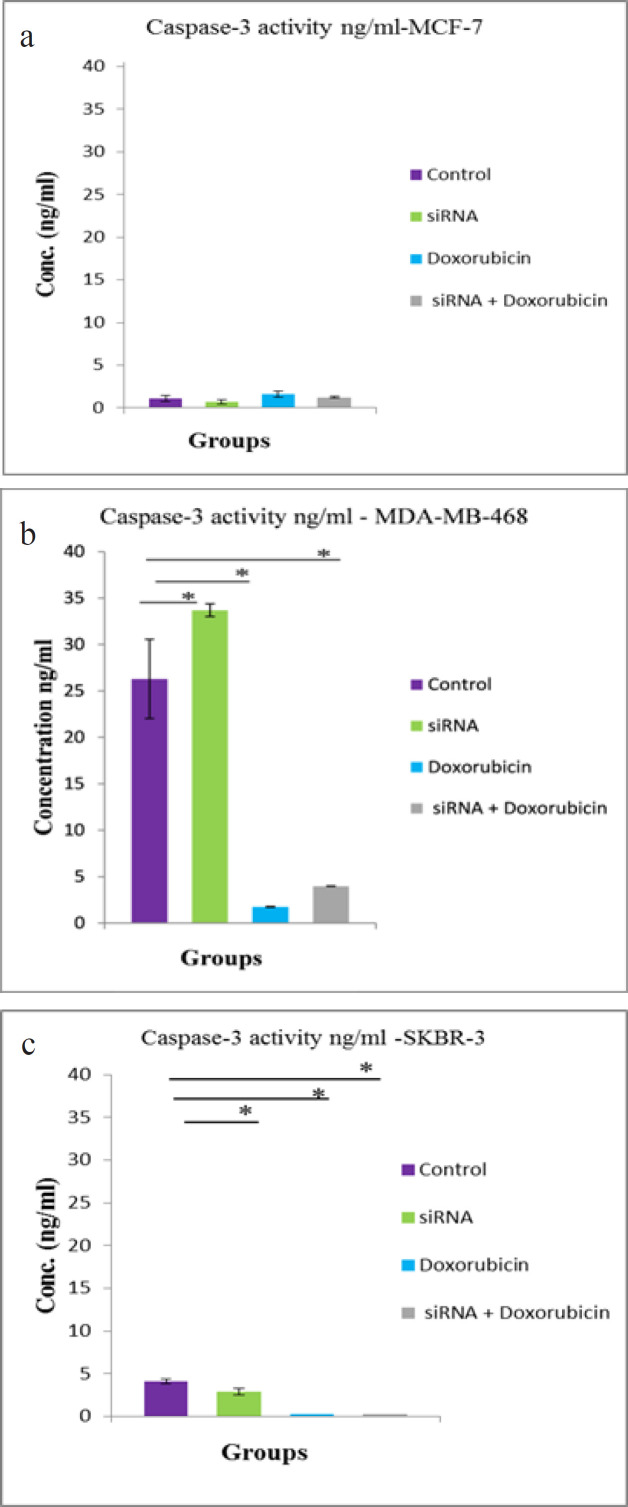
Caspase-3 Activity in a) MCF-7 b) MDA-MB-468 and c) SKBR-3 72 hours after exposure to hTERT siRNA and doxorubicin. The difference between means was carried out using one-way analysis of variance (ANOVA). Data are shown as mean ± SD (error bar) of 3 experiments, * data are significant at P < 0.05

In Figure 5, the MCF-7 cell line (Figure 5a) almost lacked caspase-3 activity in all treated groups in addition to the control group, whereas in the MDA-MB-468 cell line (Figure 5b), there was a significant increase (P < 0.003) in caspase-3 activity in the hTERT siRNA-transfected group, however, there was a significant reduction in caspase-3 activity in the doxorubicin-treated and the combined treated groups (P < 0.001), compared with the control group. The SKBR-3 cell line showed a minimal caspase-3 activity in the siRNA-transfected group compared with the control group (Figure 5c).

*Detection of morphologic changes by SEM*

**Figure 6:**
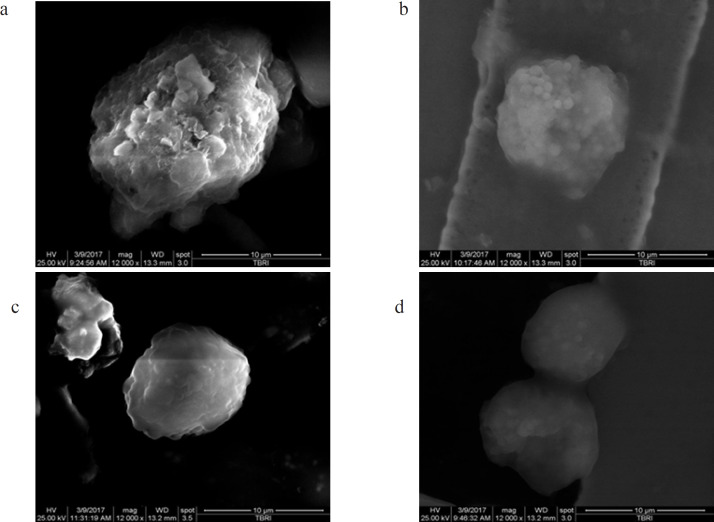
Morphologic Changes Detected by SEM in MCF-7 Cell Line. a) MCF-7 control group possessed numerous microvilli and lamellipodia on the cell surface with intact cell membrane connection that facilitate adherence of the cells. b) hTERT siRNA transfected group showed a decreased number of microvilli with a striking collection of blebs (apoptotic bodies) on the cell surface and cell shrinkage. c) Cells treated with siRNA + Doxorubicin exhibited a decrease in the number of microvilli which became relatively smooth with no obvious microvilli and blebbing on the surface of cancer cells, meanwhile progressive cell shrinkage was detected. d) Cells treated with doxorubicin showed reduction in the number of microvilli

Figure 6 (a, b, c, and d) shows the morphologic changes encountered by the three BC cell lines, represented by MCF-7, undergoing different treatment phases examined through SEM. 

## Discussion

Following telomerase activity inhibition, a synergistic process was observed based on two mechanisms; namely, DNA damage (drug-induced) and mismatch repair/genome stabilization attenuation (telomerase inhibition) (Saraswathy and Gong, 2014). The present study showed that telomerase inhibition by siRNA can be a functional treatment for BC either alone or in combination with chemotherapeutic agents. Herein, we demonstrated how the inhibitory hTERT siRNA and doxorubicin have synergistically potentiated the interruption in cell survival and growth progression of the BC cell lines in a cell-type-dependent response. In the MCF-7 and MDA-MB-468 cell lines, maximum inhibition in telomerase activity was detected in siRNA-transfected and combined treated groups; whereas in the SKBR-3 cell line, the combined treated group showed the utmost inhibition in telomerase activity. Moreover, cell viability was greatly reduced in the three BC cell lines in the combined treated group; however, in the SKBR-3 cell line, viability in the siRNA-transfected group showed a major increase, which may be due to the up-regulatory effect of HER2/neu on telomerase expression, as reported by Goueli et al., (2004) and Papanikolaou et al., (2009), who stated that hTERT expression was increased in HER2/neu positive compared with HER2/neu negative BC cell lines, where it is responsible for various biological processes, including proliferation, migration, differentiation, and apoptosis. Arman et al., (2014) claimed that while there is an increase in expression of HER2/neu in different cancer cell lines, there also is a corresponding reduction in caspase-3 / and -8 activities, thus promoting cell survival. Furthermore, Liang et al., (2001) and Yang et al., (2007) observed that receptors, such as epidermal growth factor receptor and estrogen receptors, activated telomerase through direct and indirect effects on the TERT promoter, which suggested the hormonal control of telomerase activity. In the present study, characteristic morphologic changes in the BC cells were examined through SEM. The morphologic changes were observed in the form of cell clumping and apoptotic body formation, in addition to membrane blebbing, which is part of a series of distinctive morphologic events during apoptosis. It has been reported to be associated with cytoplasmic and nuclear apoptotic manifestations, used as morphologic criteria to identify apoptosis (Majno, 1995), indicating the effects of both treatments (doxorubicin and siRNA). Rubis et al., (2013) showed that blocking telomerase activity could be used as an effective strategy to induce BC cell deaths and that cancer treatment using telomerase inhibitors will not have to be long-term and will not have to directly depend on replicative senescence and telomere length. It seems that only TERT down regulation starts the entire pathway of inducing apoptosis and cancer cell elimination. 

Caspase activation has emerged as the central molecular event leading to apoptosis, preceding DNA degradation and apoptotic morphology (Saraste, 1999). Studies of human BCs indicated an approximately 75% lack of caspase-3 transcription and protein expression (Devarajan et al., 2002). The different cell lines used in the present study showed nearly no effect on caspase-3 activation neither in control nor in the treatment groups, suggesting that caspase-3 was not involved in the apoptotic pathway under the effects of the combined treatment of hTERT siRNA and doxorubicin. On another note, caspase-8 activity slightly increased in the BC cell lines, except in SKBR-3 that showed a decrease in caspase-8 activity following treatment, indicating that the combined treatment may induce apoptosis, even in cells lacking caspases. 

This study suggests that in the three BC cell lines, responses to the combined treatment of hTERT siRNA and doxorubicin or either of them alone depends on the hormonal status of the cell lines and induction of cell death was in a caspase-8 and -3 independent manner. This might be related to estrogen and progesterone expression in cells, which was present in the MCF-7 cell line, but absent in the MDA-MB-468 and SKBR-3 cell lines. This was in accordance with Aghababazadeh et al., (2017), who showed that caspase-8 expression was significantly correlated with hormonal receptors’ status (estrogen and progesterone). Yang et al., (2001) reported that the genomic deletion of the caspase-3 gene in MCF-7 cells leads to the inability to carry out normal apoptosis and offered a possible mechanism to trigger chemoresistance. The MCF-7 cells lacking caspase-3 underwent a caspase-independent manner of apoptotic cell death, with other apoptosis executioners, such as caspase-7, that acts as a func­tionally compensative candidate for caspase-3. Liang (2001) reported that MCF-7 cells undergo apoptosis by sequential activation of caspases-7, -9 and -6. Particularly, caspase-3 is more critical for inducing certain apoptotic events, such as DNA fragmentation. The respective initiators, caspases-8 and -9, function to activate downstream caspases-3 and -7 (Nicholson, 1999). This appears to be the major pathway for telomerase-mediated resistance to apoptosis, which was likewise reported by Yamada et al., (2003), who showed that hTERT overexpression suppressed caspase-3 activity in K562 hematopoietic cells. Luiten et al., (2003) reported the reduced caspase-3 activation after ectopic hTERT expression in T-cells; however, there have been reports of telomerase-mediated resistance to apoptosis through caspase-3-independent means, which highlight the potential complexity of telomerase-mediated resistance to apoptosis (Ren et al., 2001). The results of caspase-8 activity in doxorubicin and the combined treatment groups of the MCF-7 cells, wherein caspase-8 activity was elevated, were in accordance with Abd Razak et al., (2019), who reported that caspase activity in eupatorin-treated MCF-7 cells demonstrated caspase -8 and -9 activation in MCF-7 cells after 48 hours treatment with eupatorin. Hence the co-delivery of siRNA and anti-cancer drugs permits a specific amount of siRNA and anti-cancer drugs, at a designated ratio to be simultaneously delivered into the same cancer cell population, thereby, creating synergistic effects (Saraswathy and Gong, 2014; Ahmadzada, 2018). 

This study revealed that transfection with specific siRNA-targeting hTERT gene can inhibit BC cell proliferation and sensitize cancer cells to chemotherapy, which demonstrated the ability of telomerase inhibition as an effective treatment of BC when used alone or in combination with chemotherapeutic agents. Agents targeting the enzyme could work against a broad spectrum of tumors, since telomerase is considered a universal cancer marker. 
